# DNA barcodes using a double nanopore system

**DOI:** 10.1038/s41598-021-89017-6

**Published:** 2021-05-07

**Authors:** Swarnadeep Seth, Aniket Bhattacharya

**Affiliations:** Department of Physics, University of Central Florida, Orlando, FL 32816-2385 USA

**Keywords:** Computational biophysics, Nanoscale biophysics

## Abstract

The potential of a double nanopore system to determine DNA barcodes has been demonstrated experimentally. By carrying out Brownian dynamics simulation on a coarse-grained model DNA with protein tag (barcodes) at known locations along the chain backbone, we demonstrate that due to large variation of velocities of the chain segments between the tags, it is inevitable to under/overestimate the genetic lengths from the experimental current blockade and time of flight data. We demonstrate that it is the tension propagation along the chain’s backbone that governs the motion of the entire chain and is the key element to explain the non uniformity and disparate velocities of the tags and DNA monomers under translocation that introduce errors in measurement of the length segments between protein tags. Using simulation data we further demonstrate that it is important to consider the dynamics of the entire chain and suggest methods to accurately decipher barcodes. We introduce and validate an interpolation scheme using simulation data for a broad distribution of tag separations and suggest how to implement the scheme experimentally.

## Introduction

The use of digitized DNA-barcodes^1,2^ in species identification^3–5^ has been a standard technique for the preservation of Earth’s biological diversities^6^. The extinction of species is not homogeneous across the globe, rather a strong function of location. Many species in tropical countries are declining rapidly being on the verge of extinction. The use of portable desktop equipment will be beneficial to carry out the tests locally in different countries bypassing the restrictions of bringing samples from one country to a laboratory located in another country. Nanopore based sequencing methods, such as, MinIon produced by Oxford nanopore^7^ is an important step towards that goal which will eventually replace traditional Sanger’s type of sequencing. Thus there is a genuine need to develop real-time on site desktop methods for in-situ but fast and accurate determination of genetic information contained in barcodes. Besides, there is a broad interest in applications of nanopore sensing beyond DNA-sequencing and barcode determinations, such as, discrimination, post-translational modifications, and unfolding of various proteins^8–12^.

**Figure 1:**
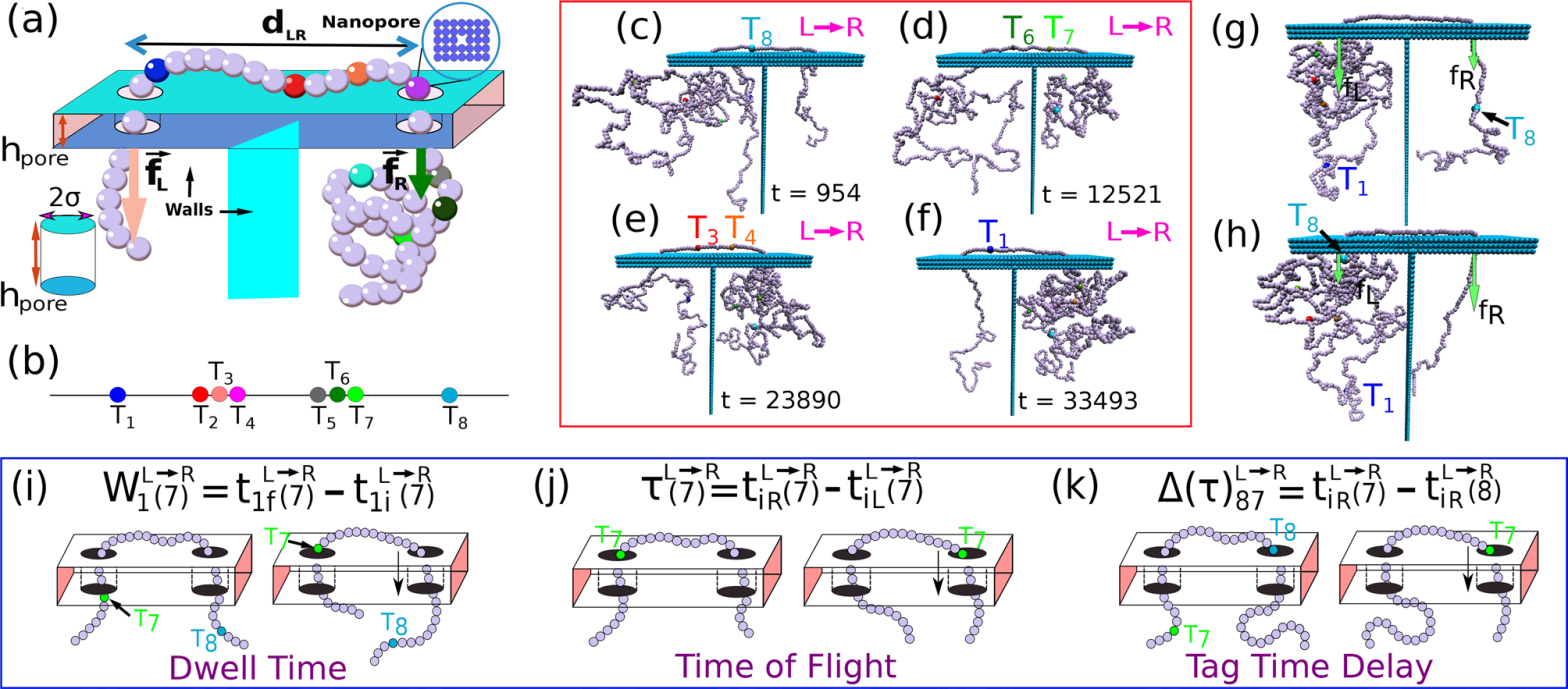
(**a**) Schematic diagram of a dsDNA captured in between two nanopores drilled through an infinitely extended material with thickness $h_{pore}$ and at a separation distance $d_{LR}$. External bias forces $\vec {f}_L$ and $\vec {f}_R$ are applied to the monomers in the left and right nanopore respectively. The colored beads are tags attached to the nucleotides and have different mass and solvent friction different from the rest of the monomers. Keeping the tug of war force at a fixed value, the force $\vec {f}_L$ and $\vec {f}_R$ are applied and varied so that DNA in between the pore has a bias $\Delta \vec {f}_{LR} = \vec {f}_L - \vec {f}_R$ to floss it from one pore to the other. (**b**) Positions of the protein tags along the dsDNA. Figures (**c**)–(**f**) show the translocation of the tags from $L \rightarrow R$ direction during scanning. The progressive uncoiling of the dsDNA contour length at the *cis* side and recoiling at the *trans* side due to the tension propagation (as observed from the simulation trajectories and the movies) are important elements to be considered to calculate the barcodes accurately (Supplementary Fig. S2). (**g**) Demonstration of flossing the DNA keeping it captured in the double nanopore system. The configuration shows a translocation taking place from $R \rightarrow L$ direction so that $\Delta \vec {f}_{LR} > 0$ and the last tag is on the right chamber. (**h**) Shows a snapshot a while later when the last tag has completed the translocation through both the pores, at which point the differential voltage is reversed so that $\Delta \vec {f}_{RL}>0$ and the translocation proceeds in the $L \rightarrow R$ direction. (**i**) Dwell time of $\mathbf{T}_{\mathbf{7}}$ is calculated by recording the time difference between arrival $t_{1i}^{L \rightarrow R} (7)$ and exiting time $t_{1f}^{L \rightarrow R} (7)$ within the left nanopore thickness for $L \rightarrow R$ translocation (see Eq. 1). (**j**) Illustration depicts the time of flight (*tof*) of $\mathbf{T}_{\mathbf{7}}$ is measured as the time taken to reach to right pore from left pore for $L \rightarrow R$ motion. (**k**) Demonstration of calculation of tag time delay $\Delta (\tau )_{87}^{L \rightarrow R}= t_{iR}^{L \rightarrow R}(7) - t_{iR}^{L \rightarrow R}(8)$ for tags $T_7$ and $T_8$ at the right pore. The similar quantity for $R \rightarrow L$ translocation $\Delta (\tau )_{78}^{R \rightarrow L}= t_{iL}^{R \rightarrow L}(8) - t_{iL}^{R \rightarrow L}(7) \ne \Delta (\tau )_{87}^{L \rightarrow R}$ as of the asymmetric tag positions along the chain. Figure (**c**)–(**h**) are generated using VMD^26^. Please also refer to the movie in the Supplemantary Information.

A double nanopore platform Fig. 1a has demonstrated that it has the ability to outperform single nanopore based devices^13,14^ in controlling the velocity and chain conformations during the translocation process. The captured DNA by both the pores is not only straightened by the tug-of-war forces present at each pore, but adjustable differential biases and feedback mechanism at each pore offers overall a better control on the translocation speed^15^. The most noteworthy aspect is the ability of multiple scans^13,16^ of the translocating DNA through the pores by flipping the net differential bias that not only increases the statistical accuracy of measurements, but in principle capable of providing additional information about the physical processes, which to date are largely unknown, and requires a thorough theoretical investigation.

While a double nanopore system offers immense promises, preliminary experiments reveal that extracting genomic lengths can be complicated due to lack of experimental information about the entire chain. The experiments can extract information about the dwell time of the protein tags in each pore and the time of flight (TOF) from one pore to the other only (Fig. 1). These information would be sufficient to extract spacing between the barcode accurately if the entire chain was moving with the same velocity. As the protein tags experience larger frictional drags, it is expected that different parts of the chain will not move through the double nanopore system with uniform velocity which will then introduce error in genomic length determination. The dispersion in velocity will also depend on the magnitude of the differential bias forces ($\Delta \vec {f}_{LR}$), the pore width ($h_{pore}$) and the distance between the two nanopores ($d_{LR}$). Experimentally nanoscale technology poses challenges to vary these parameters and can often be expensive. However, these dependencies can be efficiently studied in a model system using computer simulations strategies^17–19^ combined with theoretical methods developed in the last decade for studying translocating chain through nanopores^20–23^ and validated by simulation studies^24,25^.

**Figure 2:**
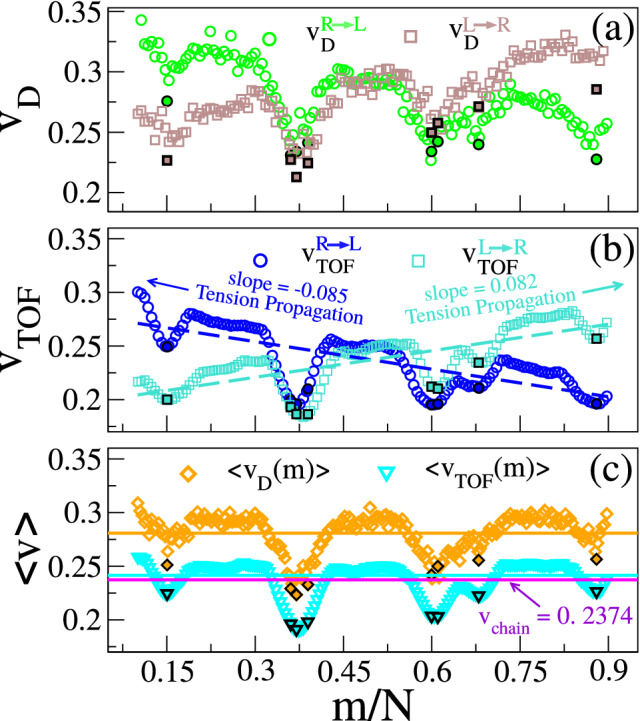
(**a**) Dwell velocities and (**b**) TOF velocities of the monomers during $R \rightarrow L$ (blue circle) and $L \rightarrow R$ (turquoise square) translocation. Eight tag velocities are marked by the filled symbols. The dashed lines (turquoise and blue) indicate the directions of the tension propagation for $L \rightarrow R$ and $R \rightarrow L$ respectively. (**c**) Directional averaged dwell velocity and TOF velocities are represented with orange diamond and turquoise inverted triangle. The solid orange horizontal line represents average dwell velocity and the cyan line represents the average tof velocity of all monomers respectively. The average velocity of the entire chain is represented by the violet line.

In this article, we present Brownian dynamics simulation results for a coarse-grained model of dsDNA with protein tags attached to it mimicking the essentials of the experimental setup. The simulation data shows that the velocities of the chain segments are indeed nonmonotonic as a function of the monomer index (Fig. 2). We further demonstrate that using the information about only the protein tags to extract barcode distances, as measured in an experiment, lead to an over/underestimation of the barcode distances. We then use the nonequilibrium tension propagation (TP) theory of Sakaue^23^ to explain the non uniformity of the velocity profile. The underlying physical picture that emerges also provides clues for the under/over estimation of the barcode distances and direct us to an interpolation scheme to determine the barcode distances accurately.

## Coarse-grained model and Brownian dynamics

Our coarse grained model consists of a polymer chain of 1024 beads with 8 protein tags translocating through a double nanopore system (Fig. 1a), inspired by the 48 kbp long double-stranded $\lambda$-DNA used in the experiment by Liu et al.^16^, where sequence-specific protein tags are introduced chemically so that the distance between any two tags (Fig. 1b) is known.

In the simulation, each bead (monomer) represents approximately 46 bp unit long dsDNA and one tag is roughly equivalent to 75 bp in the experiment which translates to approximately 2–3 beads. The mapping procedure is discussed in the Supplementary Information and the general scheme of the BD simulation strategy for a homopolymer translocating through a double nanopore has been discussed in our recent publication^17,18^. The protein tags are introduced by choosing the mass and friction coefficient at tag locations to be significantly different from the rest of the monomers along the chain (which requires modification of the BD algorithm as discussed in the Supplementary Information I and Supplementary Fig. S1). Instead of explicitly putting side-chains at the tag locations, we made the mass and the friction coefficient of the tags 3 times larger. Typically heavier biomolecules in a solvent are correlated with larger volumes which would produce larger drags. The volume effect is implicit in our scheme as this to a first approximation will renormalize the friction. This we find enough to resolve the distances among the tags. The location of the tags are also chosen in such a way so that genetic distances are disparate (some tags being close by and some are far apart) as shown in Fig. 1b and Table 1. It is also noteworthy that there is no left to right symmetry so that the center of mass of the chain is not located at the center of the chain. The key question to answer is mimicking the double nanopore experimental protocol how accurately can one extract these genomic distances so that the method then can be applied to determine genetic lengths in unknown specimens.

**Table 1:** Tag positions along the dsDNA.

Tag	$T_1$	$T_2$	$T_3$	$T_4$	$T_5$	$T_6$	$T_7$	$T_8$
Position	154	369	379	399	614	625	696	901
Separation	154	215	10	20	215	11	71	205

## Repeated scans and measurements

The measurement protocols are described in Fig. 1g–j. The differential bias $\Delta \vec {f}_{LR}=\vec {f}_{L}-\vec {f}_R > 0$ for the $R \rightarrow L$ translocation (Fig. 1g). Once the last tag passes through the right pore, the bias is switched to $\Delta \vec {f}_{RL}=\vec {f}_{R}-\vec {f}_L > 0$ (Fig. 1h) so that the direction of translocation is reversed. Here, we report the case for $|\Delta \vec {f}_{LR}|=|\Delta \vec {f}_{RL}|$. Later we mention what happens when an asymmetry is present and $|\Delta f_{LR}| \ne |\Delta f_{RL}|$. This process (called flossing—one flossing consists of one $R \rightarrow L$ and one $L \rightarrow R$ scan) is repeated 300 times and we record the experimentally measurable quantities—the dwell time and the time of flight (*tof*) as described next. We reserve the subscripts 1 and 2 for the left pore (pore 1) and right pore (pore 2) respectively. For sake of brevity we define quantities for the $L \rightarrow R$ only, implicating that DNA is translocation from left pore to right pore and the same definitions hold for the $R \rightarrow L$ translocation.

### Dwell time

The co-captured dsDNA in a dual nanopore system provides two different ways of time measurements for the protein tags during translocation which can be translated to genomic lengths. Similar to a single nanopore setup, one can measure the *dwell time* in each pore for a double nanopore setup. For example, for the $L\rightarrow R$ translocation (Fig. 1i), one can measure the dwell time by recording the arrival $t_i^{L \rightarrow R}(m)$ and the exit time $t_f^{L \rightarrow R}(m)$ of a monomer *m* at the left (1) or the right (2) pore. For the $L \rightarrow R$ and $R \rightarrow L$ translocation directions the dwell time at the left pore is defined as follows:

1a $$\begin{aligned} W_1^{L \rightarrow R}(m)= & {} t_{1f}^{L \rightarrow R}(m) - t_{1i}^{L \rightarrow R}(m), \end{aligned}$$

1b $$\begin{aligned} W_1^{R \rightarrow L}(m)= & {} t_{1f}^{R \rightarrow L}(m) - t_{1i}^{R \rightarrow L}(m). \end{aligned}$$

Likewise, $W_2^{L \rightarrow R}(m)$ and $W_2^{R \rightarrow L}(m)$ can be obtained replacing 1 by 2 in the above equation. An example of dwell time calculation for ${T}_{\mathbf{7}}$ is shown in Fig. 1i. The dwell time is the quantity which can be compared with the experimental duration of the *current blockade time* of the monomers and the protein tags.

### The time of flight (tof)

In addition to the dwell time measurements, in a double nanopore setup, one can also measure the time taken by a monomer as it leaves one pore and touches the upper boundary of the other pore during its voyage across the pore separation $d_{LR}$ (Fig. 1j). This is called the time of flight *tof* and defined as follows:

2a $$\begin{aligned} \tau ^{L \rightarrow R}(m)= & {} t_{iR}^{L \rightarrow R}(m) - t_{iL}^{L \rightarrow R}(m), \end{aligned}$$

2b $$\begin{aligned} \tau ^{R \rightarrow L}(m)= & {} t_{iL}^{R \rightarrow L}(m) - t_{iR}^{R \rightarrow L}(m). \end{aligned}$$

### Dwell velocity $v_D$ and time of flight velocity $v_{tof}$

Accordingly, one can calculate both $v_D$ as well as $v_{tof}$ using Eqs. (1) and (2) as follows,

3a $$\begin{aligned} v_{D}^{L \rightarrow R}(m)= & {} \frac{1}{2}\left[ \frac{h_{pore}}{W_1^{L \rightarrow R}(m)} + \frac{h_{pore}}{W_2^{L \rightarrow R}(m)} \right] , \end{aligned}$$

3b $$\begin{aligned} v_{D}^{R \rightarrow L}(m)= & {} \frac{1}{2}\left[ \frac{h_{pore}}{W_1^{R \rightarrow L}(m)} + \frac{h_{pore}}{W_2^{R \rightarrow L}(m)} \right] , \end{aligned}$$

3c $$\begin{aligned} \langle v_D(m) \rangle= & {} \frac{1}{2}\left( \langle v_{D}^{L \rightarrow R}(m) \rangle + \langle v_{D}^{R \rightarrow L}(m) \rangle \right) , \end{aligned}$$

4a $$\begin{aligned} v_{tof}^{L \rightarrow R}(m)= & {} d_{LR}/\tau ^{L \rightarrow R}(m), \end{aligned}$$

4b $$\begin{aligned} v_{tof}^{R \rightarrow L}(m)= & {} d_{LR}/\tau ^{R \rightarrow L}(m), \end{aligned}$$

4c $$\begin{aligned} \langle v_{tof}(m) \rangle= & {} \frac{1}{2}\left( \langle v_{tof}^{L \rightarrow R}(m) \rangle + \langle v_{tof}^{R \rightarrow L}(m) \rangle \right) . \end{aligned}$$

Here $\langle \cdots \rangle$ implies average over multiple scans through the left and the right pore which reduces the statistical error in the measurements.

## Results

### Non uniform velocity profile

Due to lack of $L \rightarrow R$ symmetry both $v_{D}$ and $v_{tof}$ have a positive slope along the direction of translocation due to propagation of the tension front^24^ (Fig. 2a,b). Figure 2c shows that average of both the directions and the slope is close to zero as we are considering the symmetric differential bias $|\Delta \vec {f}_{LR}|=|\Delta \vec {f}_{RL}|$. As expected, the velocity distribution of the chain segments become non-monotonic with respect to the average velocity of the entire chain. The protein tags with heavier mass ($m_{tag} > m_{bulk}$) and larger solvent friction ($\gamma _{tag} > \gamma _{bulk}$) reside at the lower envelope of the graphs (Fig. 2c) while the dsDNA monomers reach their maximum velocity somewhere in between the tags. In addition to the average dwell and time of flight velocities $\langle v_{D}\rangle$ and $\langle v_{tof}\rangle$, we have plotted the average velocity of the entire chain $\langle v_{chain}\rangle$, each represented as a solid line. For the choice of the parameters the dwell time velocities are larger and $\langle v_{D}\rangle$ is about 20% larger than the $\langle v_{tof}\rangle$. This is a coincidence and not a generic feature. We have checked that keeping all the parameters the same, an increase in pore thickness $h_{pore}$ will result in an overall decrease of the dwell time velocities due to increased friction for a thicker pore. It is also worthwhile to note that although in simulation we can calculate the dwell time and time of flight for all the monomers and tags, experimentally these data (Eqs. 1 and 2) are measured for the protein tags only as the tags produce significant current blockades to be measured. However, the entire chain contributes to the dynamics of the tags, and it is this lack of information for the entire chain inevitably leads to inaccurate measurements of the genomic length as demonstrated below.

### Barcodes from the segmental velocities

Let us first evaluate the consequence if we calculate the average velocity of the segment $v_{seg}^{L \rightarrow R} (m,n)$ connecting the tags $T_m$ and $T_n$ by approximating it to be the average velocity of the tags $T_m$ and $T_n$ only so that

5a $$\begin{aligned}&v_{seg}^{L \rightarrow R} (m,n) \approx \frac{1}{2} \left[ v_{tof}^{L \rightarrow R}(m) + v_{tof}^{L \rightarrow R}(n)\right] , \end{aligned}$$

5b $$\begin{aligned} v_{seg}^{R \rightarrow L} (m,n) \approx \frac{1}{2} \left[ v_{tof}^{R \rightarrow L}(m) + v_{tof}^{R \rightarrow L}(n)\right] . \end{aligned}$$

Barcode distances $d_{tof}^{L \rightarrow R} (m,n)$ and $d_{tof}^{R \rightarrow L} (m,n)$ between $T_m$ and $T_n$ for $L(R) \rightarrow R(L)$ translocations are then obtained as

6a $$\begin{aligned} d_{tof}^{L \rightarrow R} (m,n)&\approx v_{seg}^{L \rightarrow R}(m,n) \times (\Delta \tau )_{mn}^{L \rightarrow R}, \end{aligned}$$

6b $$\begin{aligned} d_{tof}^{R \rightarrow L} (m,n)&\approx v_{seg}^{R \rightarrow L} (m,n) \times ( \Delta \tau )_{mn}^{R \rightarrow L}, \end{aligned}$$

6c $$\begin{aligned} d_{tof}(m,n)&\approx \frac{1}{2}\left( d_{tof}^{L \rightarrow R} (m,n) + d_{tof}^{R \rightarrow L} (m,n) \right) . \end{aligned}$$

Here, $(\Delta \tau )_{mn}^{L \rightarrow R}$ and $(\Delta \tau )_{mn}^{R \rightarrow L}$ are the time difference of arrival (we call it *tag-time-delay*) of the *m*-th and the *n*-th tags at *L*/*R* pore during $R/L \rightarrow L/R$ translocation. Fig. 1k illustrates a specific case $(\Delta \tau )_{87}^{L \rightarrow R}$. Equation 6c provides the final distance $d_{tof}(m,n)$ averaged over multiple scans in each direction. Likewise, using dwell time velocity Eqs. (3a–3c), and replacing the subscript *tof* by *D* in Eqs. (5a,5b), one can derive equations analogous to Eqs. (6a–6c). A similar equation for the barcode distance using dwell time data from both the pores:

7 $$\begin{aligned} d_{dwell}(m,n) \approx \frac{1}{2}\left( d_{dwell}^{L \rightarrow R} (m,n) + d_{dwell}^{R \rightarrow L} (m,n) \right) . \end{aligned}$$

**Figure 3:**
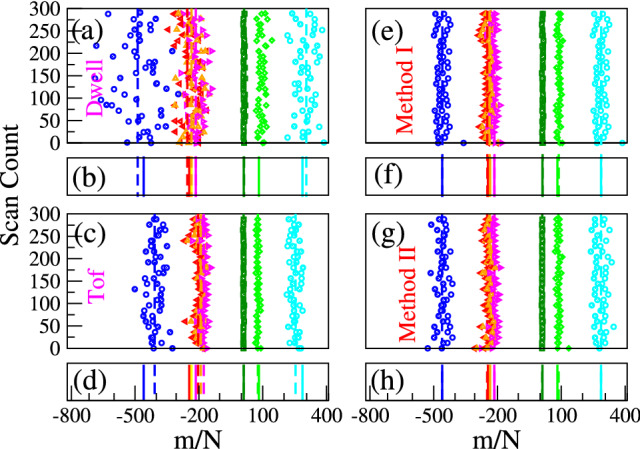
Barcodes generated using (**a**) Eq. (7) and (**c**) Eq. (6c). Colored symbols/lines refer to the barcodes $T_1$, $T_2$ , $T_3$, , $T_4$, $T_6$, $T_7$, and $T_8$ calculated w.r.t $T_5$. The open/filled symbols correspond to $R/L\rightarrow L/R$ translocation. For better visualization, every 6th data point is shown. The solid/dashed colored lines in (**b**) and (**d**) are the exact and the calculated barcodes averaged over 300 scans. Figures (**e**,**f**) Barcodes generated using different methods using tof velocity information.

The distribution of barcode distances with respect to ${T}_{\mathbf{5}}$ using Eqs. (6c) and (7) are shown in Fig. 3a–d and summarized in the 4th and 3rd columns of Table 2 respectively.

**Table 2:** Velocity to Barcodes using different methods.

Tag	Relative	Barcode	Barcode	$v_{D}$	$v_{tof}$	$v_{chain}$	Barcode	Barcode	Barcode
Label	Position	Dwell	Tof	($10^{-1}$)	($10^{-1}$)	$\simeq {\bar{v}}_{scan} (10^{-1})$	Method I	Method II	Method II
Figure 2	Exact	Eq. (7)	Eq. (6c)	Eq. (3c)	Eq. (4c)	Eq. (10)	Dwell/Tof	Dwell	Tof
$T_1$	− 460	$-486 \pm 124$	$-407 \pm 45$	$2.59 \pm 0.87$	$2.21 \pm 0.34$	$2.37 \pm 0.16$	$-459 \pm 17$	$-461 \pm 27$	$-461 \pm 27$
$T_2$	− 245	$-251 \pm 64$	$-203 \pm 25$	$2.32 \pm 0.83$	$1.92 \pm 0.30$		$-248 \pm 16$	$-249 \pm 19$	$-248 \pm 19$
$T_3$	− 235	$-237 \pm 60$	$-191 \pm 24$	$2.31 \pm 0.75$	$1.87 \pm 0.27$		$-236 \pm 16$	$-237 \pm 18$	$-237 \pm 18$
$T_4$	− 215	$-219 \pm 57$	$-177 \pm 22$	$2.37 \pm 0.83$	$1.94 \pm 0.28$		$-213 \pm 16$	$-214 \pm 17$	$-214 \pm 17$
$T_5$	0	0	0	$2.51 \pm 0.90$	$2.00 \pm 0.28$		0	0	0
$T_6$	11	$12 \pm 3$	$10 \pm 1$	$2.55 \pm 0.87$	$2.00 \pm 0.29$		$12 \pm 2$	$11 \pm 2$	$10 \pm 2$
$T_7$	82	$93 \pm 23$	$77 \pm 7$	$2.63 \pm 0.93$	$2.19 \pm 0.35$		$87 \pm 10$	$87 \pm 11$	$87 \pm 11$
$T_8$	287	$304 \pm 72$	$254 \pm 26$	$2.62 \pm 0.91$	$2.23 \pm 0.31$		$287 \pm 17$	$287 \pm 27$	$287 \pm 26$

A closer look reveals the over/under estimation of the barcodes (columns 3 and 4) w.r.t the theoretical value (column 1) occurs when $v_D$ and $v_{tof}$ (columns 5 and 6) are greater or less than the average velocity of the entire chain $\langle v_{chain}\rangle$ (column 7), and can be immediately discerned from Fig. 2. Furthermore this is an *uncontrolled* velocity approximation introduced in Eqs. (5a,5b) and depends on the contour length separation between the tags which is the unknown and yet to be determined. We further observe that since $v_{tof}(m) < v_{chain}$ for all the tags $m=1, 8$, the barcode distances are underscored. On the contrary, $v_D(m)$ for the tags are more dispersed above and below $v_{chain}$, and whenever $v_D(m) \simeq v_{chain}$, Eq. (7) gives a better agreement (for $\mathbf{T}_\mathbf{3}$ and $\mathbf{T}_\mathbf{4}$). If we replace the approximate velocities in Eq. (6c) by the constant velocity $v_{chain}$ of the entire chain this improves the estimates significantly. This is shown in column 8 (Barcode Method-I) and discussed later. We now explain the source of discrepancy using the non-equilibrium TP theory.

#### TP Theory explains the source of discrepancy and provides a solution

Unlike a rigid rod, tension propagation has a considerable effect in governing the motion of a semi-flexible chain in the presence of an external bias force. In TP theory^23^ and its implementation in Brownian dynamics^24,25^ the motion of the subchain in the *cis* side decouples into two domains. In the vicinity of the pore, the tension front affects the motion directly while the second domain remains unperturbed, beyond the reach of the TP front.

In our case, after the tag $T_m$ translocates through the pore, following monomers are dragged into the pore quickly by the tension front, analogous to the uncoiling effect of a rope pulled from one end (Please refer to Supplementary Fig. S2 and the movie in the Supplementary Information). The onset of this sudden *faster* motion continues to grow (Supplementary Fig. S2a) and reaches its maximum until the tension front hits the subsequent tag $T_{m \pm 1}$ (for $R/L \rightarrow L/R$ translocation direction), having larger inertia and viscous drag. At this time (called the tension propagation time^25^) the faster motion of the monomers begins to taper down to the velocity of the tag $T_{m \pm 1}$. An example of this process is shown in Supplementary Fig. S2b. This process continues from one segment to the other and explains oscillatory characteristics in Fig. 2c. These contour lengths of faster moving segments in between two tags are accounted for neither in Eq. (6c) nor in Eq. (7). The experimental protocols are limited in extracting barcode information through Eqs. (6c) and (7) (measuring current blockade time) and could be the possible source of error.

### How to estimate the barcodes accurately?

We now propose two methods that take into account the dynamics of the entire chain and correctly determine the barcodes and can be implemented in a dual nanopore setup experimentally.

#### Method 1: Barcode from known end-to-end tag distance

If the distance between the first tag $T_1$ and the last tag $T_8:$ $d_{18}\simeq L$, then the velocity of the segment $d_{18}$ will approximately account for the average velocity of the entire chain ($v_{chain}$) so that

8 $$\begin{aligned} v_{chain}^{L \rightarrow R} \approx v_{18}^{L \rightarrow R} = d_{18}/(\Delta \tau )_{18}^{L \rightarrow R}, \end{aligned}$$

assuming we know $d_{18}$ and $(\Delta \tau )_{18}^{L \rightarrow R}$ is the time delay of arrival at the pore between $T_1$ and $T_8$ for ${L \rightarrow R}$ translocation. We then estimate the barcode distance $d_{mn}^{L \rightarrow R}$ between tags $T_m$ and $T_n$ as

9 $$\begin{aligned} d_{mn}^{L \rightarrow R} = v_{18}^{L \rightarrow R} \times (\Delta \tau )_{mn}^{L \rightarrow R}. \end{aligned}$$

The barcodes for the $L \rightarrow R$ and $R \rightarrow L$ translocation are shown in Fig. 3e. The average shown in Fig. 3f corresponds to column 8 of Table 2 as mentioned earlier. This is a significant improvement compared to the usage of the average tag velocity of the chain segments. This method will work if one can put two additional tags at known distances, at or close to the two ends of the DNA being scanned. Alternately, scan time information can be used to have a better estimate of the average velocity of the chain. In our simulation, we kept the scanning length $L_{scan}$ constant with starting and ending values ranging from 0.0976*L* to 0.902*L*. By using the constant scanning length $L_{scan}$, the average scan velocity ${\bar{v}}_{scan}$ can be used to determine barcode distances by replacing $v_{chain}\simeq v_{18}$ in Eq. (9) with

10 $$\begin{aligned} {\bar{v}}_{\mathrm{scan}} = \frac{1}{N_{\mathrm{scan}}}\sum _{i=1}^{N_{\mathrm{scan}}} L_{\mathrm{scan}}/\tau _{\mathrm{scan}}(i), \end{aligned}$$

where $\tau _{\mathrm{scan}}(i)$ is the scan time for the *i*th event, and $N_{\mathrm{scan}}=300$.

#### Method 2: Barcode using two-step method

Having gained a better understanding of the velocities of the monomers of the dsDNA segments in between the tags we now rectify Eqs. (6c) and (7) by taking a *weighted average* of the velocities of tags and DNA segment in between as follows. First, we estimate the approximate number of monomers $N_{mn}\simeq d_{tof}^{L \rightarrow R}/\langle b_l \rangle$ ($\langle b_l \rangle$ is the bond length) by considering the tag velocities only using Eq. (6c). We further re-calculate the segment velocity accurately by incorporating weighted velocity contributions from both the tags and the monomers between the tags as follows:

11 $$\begin{aligned} \begin{aligned} v_{weight}^{L \rightarrow R}&= \frac{1}{N_{mn}} \Big [ n_{next} \left( v_{tof}^{L \rightarrow R}(m) + v_{tof}^{L \rightarrow R}(n) \right) \\&\quad + \left( N_{mn}-2n_{next}\right) {\bar{v}}_{\mathrm{scan}} \Big ]. \end{aligned} \end{aligned}$$

Here, $n_{next}$ are the number of neighboring monomers adjacent to the tags those share the same tag velocity. We checked that $n_{next} \approx 1--3$ does not make a noticeable difference in the final result. The barcode distances are finally calculated as

12 $$\begin{aligned} d_{mn}^{L \rightarrow R} = v_{weight}^{L \rightarrow R}\times (\Delta \tau )_{mn}^{L \rightarrow R} \end{aligned}$$

for $$L \rightarrow R$$ translocation and repeating the procedure for $$R \rightarrow L$$ translocation, shown in Fig. 3g for both $${L \rightarrow R}$$ and $${R \rightarrow L}$$ translocation. The average shown in Fig. 3h corresponds to column 10 of Table 2. It is worth noting that (i) in Eq. (11) the tag velocities are more weighted and makes a difference when $$N_{mn} $$ is small, i.e., the contour length between the tags is small, in which cases the monomers in between the tags move with almost same velocity as that of the tags. In the other limit when $$N_{mn}>>1$$, it is the chain velocity that dominates and one can safely ignore the velocity of the two tags (the 1st two terms in Eq. 11). Since the number of tags are only a few (8 in 1024 in our case), Eq. (9) works well excepting when the tags are close by. (ii) The “two step” weighting procedure in Eq. (11) is only approximate and has room for further improvement as one can interpolate from $$v_{tag}$$ to $$v_{chain}$$ with a suitable interpolation scheme.

#### Dwell time versus TOF

We have repeated the same protocol to correct the data from the dwell time measurement replacing $v_{tof}$ in Eq. (11) by $v_{D}$ listed in column 10 of Table 2. They are practically indistinguishable excepting for short tag distances.

### Tag-time-delay matrix and the sum-rule

**Figure 4:**
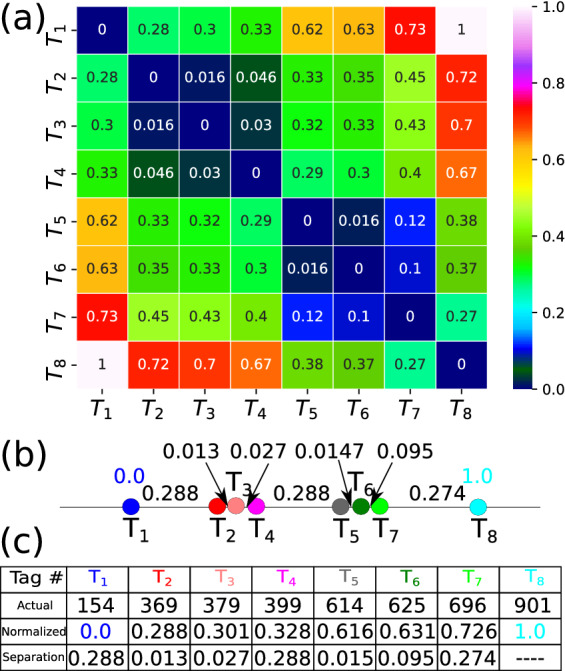
(**a**) Heat map of the normalized tag time delay $({\tilde{\Delta }}\tau )_{mn}=(\Delta \tau )_{mn} / (\Delta \tau )_{18}$ for $m=1,8$ and $n=1,8$. (**b**) Inter tag spacing in the normalized unit where $d_{18} = 1.0$. The normalized tag time delays are proportional to the normalized spacing distances validate the accuracy of Method I. (**c**) Table contains the actual tag positions and normalized inter-spacing distances.

If we use $v_{chain}=\mathrm {constant}$ to determine the barcodes as in Eq. (9), then the average *tag-time-delay* $\langle (\Delta \tau )_{mn} \rangle = \frac{1}{2} \langle (\Delta \tau )_{mn}^{L \rightarrow R} + (\Delta \tau )_{mn}^{R \rightarrow L} \rangle$ will be proportional to the barcode distances. One can then form a heat map of the normalized tag time delay $({\tilde{\Delta }}\tau )_{mn}=(\Delta \tau )_{mn}/(\Delta \tau )_{18}$ in the form of a $8\times 8$ matrix as shown in Fig. 4. The values in each square when multiplied by the appropriate scale factor ($(\Delta \tau )_{18}\times v_{chain}$) will reproduce the barcode distances and can serve as a nice visual about the relative distances between the tags. We find that indeed it reproduces barcodes of Fig. 1b as listed in Table 1.

We also find a “sum-rule” $({\tilde{\Delta }}\tau )_{mn}=({\tilde{\Delta }}\tau )_{mp}+({\tilde{\Delta }}\tau )_{pn}$ to be satisfied. As an example, let’s choose $m=1$, $n=3$ so that $({\tilde{\Delta }}\tau )_{13}= 0.30 =({\tilde{\Delta }}\tau )_{12} + ({\tilde{\Delta }}\tau )_{23} = 0.280 + 0.016 = 0.296 \simeq 0.30$, the normalized distance between the tag $T_1$ and $T_3$. In general, one can check that

13 $$\begin{aligned} ({\tilde{\Delta }}\tau )_{mn}= ({\tilde{\Delta }}\tau )_{m (m+1)}+({\tilde{\Delta }}\tau )_{(m+1) (m+2)}\cdot \cdot ({\tilde{\Delta }}\tau )_{(n-1) n}. \end{aligned}$$

Thus this *sum-rule* can be used to measure the distance between barcodes in many different ways, reduce the uncertainties, and possibly infer information about a missing tag from the self-consistency checks using Eq. (13).

#### Realistic pores and biases

Two pores in an experimental dual nanopores system are not exactly identical, about 5–10% differences in pore diameters are reported^16^. Likewise, when the differential bias is reversed $\Delta \vec {f}_{LR} = \vec {f}_L-\vec {f}_R \ne \Delta \vec {f}_{RL} = \vec {f}_R-\vec {f}_L$ for translocation directions $L \rightarrow R$ and $R \rightarrow L$ respectively. We ran additional simulations by offsetting the ideal conditions and checked that the same methods (Method I and II) work.

## Summary and concluding remarks

Dual nanopore platform has immense promise and advantages compared to its single nanopore counterpart. In this Brownian dynamics simulation study, we mimicked an experimental platform and explained why extracting information from the tags only need to be corrected by taking into account the motion of the entire chain. We invoked TP theory to explain the nonuniform velocity distribution of the entire chain as a function of the monomer index. The protein tags introduce oscillation on the uniform velocity of the chain that depends on the tension propagation time from one tag to the other. We have checked that the information obtained from the time of flight data is more accurate compared to the dwell time data from the individual pores. We further discovered that the most reliable quantity is the *tag-time-delay* of the successive barcodes to arrive at the L/R pore. When the distance between the tags is large the *tag-time-delay* will straight translate to genomic length excepting for those cases when the tags are close by. Our two-step interpolation scheme will overcome this issue. This is due to the fact that roughly it is the average velocity of the entire chain and not the average velocity of the two tags that needs to be used to calculate the barcode distances. The heat-map of the normalized *tag-time-delay* provides and the corresponding *sum-rule* are the direct proof of the efficacy of this method. This study also indicates how to improve the measurement protocol. With some prior information about the tags if one can selectively attach heavier molecules at the tag positions so that they produce sharp dips on the velocity profile of the entire chain, then the procedure will be more accurate.

We would like to make some comments on relating these results with the time scale of the actual experiment and the effects of the pores. Our coarse-grained chain length will inevitably lead to a faster translocation process in simulation compared to the actual speed of the dsDNA. It is clear from watching the simulation movies that the tension propagation occurs in the model system well within the scan time. Thus, it is expected that in the actual experiment this will be more prominent due to the slower movement of the chain. Secondly, in experiments it is likely that there are cross-talks between the forces at each pore (the authors are indebted to Walter Reisner and William Dunbar for bringing this to their attention), while in our simulation the forces are strictly local. Moreover, experimental pores are much wider, and thus folded configurations can translocate which are not included in our model with narrow pores. The tags can introduce additional pore–poymer interaction. These items will not change the main conclusion of the paper, however, will be included in our subsequent studies of polymer translocatoin through a double nanopore system in a broader context. We believe that this study will be immensely useful for designing future double nanopore platforms so that the data in the time domain can be translated to unravel fine structures of genomic lengths.

## Supplementary information

Supplementary Video.

Supplementary Information.
