# Supplementary material for: DNA barcodes using a double nanopore system
Source: Sci Rep. 2021 May 7;11:9799. doi: 10.1038/s41598-021-89017-6 (PMC8105412; doi:10.1038/s41598-021-89017-6)
Supplement: Supplementary file 2 — Supplementary Information. [file 41598_2021_89017_MOESM2_ESM.pdf]

Supplemental Materials: DNA Barcodes using a Double Nanopore System

Swarnadeep Seth[1] and Aniket Bhattacharya[1]
[1 1]*Department of Physics, University of Central Florida, Orlando, Florida 32816-2385, USA*

I. SECTION 1: THE MODEL

Our BD scheme is implemented on a bead-spring model of a polymer with the monomers interacting via an excluded volume (EV), a Finite Extension Nonlinear Elastic (FENE) spring potential, and a bond-bending potential enabling variation of the chain persistence length ℓ_p (Fig.S1). The model, originally introduced for a fully flexible chain by Grest and Kremer [S1], has been studied quite extensively by many groups using both Monte Carlo (MC) and various molecular dynamics (MD) methods [S2]. Recently we have generalized the model for a semi-flexible chain and studied both equilibrium and dynamic properties [S3–S5] and studied compression dynamics of a model dsDNA inside a nanochannel [S6, S7] . The mutual EV interaction among any two monomers are given by the truncated Lennard-Jones (LJ) potential with a cut-off radius $2^{1/6}\sigma$

$$U_{LJ}(r_{ij}) = \begin{cases} 4\epsilon\left[\left(\frac{\sigma}{r_{ij}}\right)^{12} - \left(\frac{\sigma}{r_{ij}}\right)^{6}\right] + \epsilon, & \text{for } r < 2^{1/6} \\ 0, & \text{otherwise} \end{cases} \tag{S1}$$

where σ is the effective diameter of a monomer and ϵ is the interaction strength. To mimic the connectivity between two adjacent monomers, finite-extensible-non-linear elastic (FENE) potential

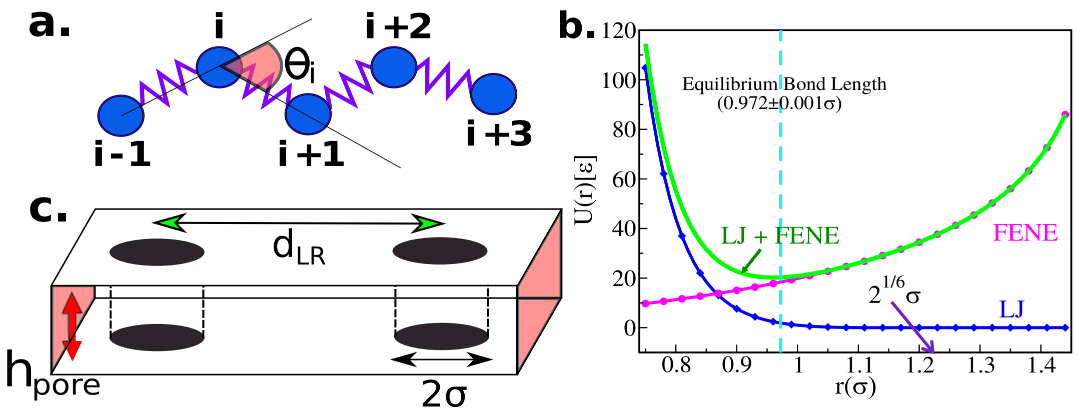

FIG. S1. (a) Illustration depicts the monomers are interacting via LJ and FENE potential. The three-body bending potential is calculated using the angle θ_i between two adjacent bond vectors $\vec{b}_i$ and $\vec{b}_{i+1}$ respectively. (b) Interaction potential between two consecutive monomers is given by the green line for a separation distance r in the unit of σ. The blue diamonds denote the LJ potential with a cutoff radius $2^{1/6}\sigma$ and the magenta circles correspond to the FENE potential with a spring constant $\kappa_F = 30.0\epsilon/\sigma^2$. (c) Two nanopores of diameter 2σ are drilled into a material of thickness h_{pore} at a separation distance $d_{LR} = 32\sigma$ between them. The purely repulsive walls consist of layers of immobile LJ particles of diameter σ (Please see Fig. S3 with three layers of wall particles).

$$U_{FENE}(r_{ij}) = -\frac{1}{2}\kappa_F R_0^2 \ln\left[1 - \left(\frac{r_{ij}}{R_0}\right)^2\right] \tag{S2}$$

is used with the maximum bond-stretching length $R_0 = 1.5\sigma$ and spring constant $\kappa_F = 30\epsilon/\sigma^2$. Here, $r_{ij} = |\vec{r}_i - \vec{r}_j|$ is the separation distance between two adjacent monomers i and $j = i \pm 1$ located at $\vec{r}_i$ and $\vec{r}_j$ respectively. Along with these two potentials, we introduce a bending potential

$$U_{bend}(\theta_i) = \kappa\left(1 - \cos\left(\theta_i\right)\right) \tag{S3}$$

with bending rigidity κ. In three dimensions, for $\kappa \neq 0$, the persistence length ℓ_p of the chain is related to κ via [S8]

$$\ell_p = \frac{\kappa}{k_B T}, \tag{S4}$$

where k_B is the Boltzmann constant and T is the temperature. Here θ_i is the bond angle between two subsequent bond vectors $\vec{b}_i = \vec{r}_{i+1} - \vec{r}_i$ and $\vec{b}_{i-1} = \vec{r}_i - \vec{r}_{i-1}$. Two nanopores of diameter 2σ are drilled through a solid material of thickness h_{pore} consists of layers of immobile repulsive LJ particles. The separation between the nanopores is kept at d_{LR} and $d_{LR}/L << 1$, L being the contour length of the dsDNA . Our model of DNA polymer consists 1016 monomer beads along with 8 heavier tags (T_1 - T_8) located at positions $154, 369, 379, 399, 614, 625, 696,$ and 901 respectively (please refer to Fig. 2 and Table-I in the main article). A recent study by Zhang et al. on 48512 bp long dsDNA uses protein tags (roughly equivalent to 75 bp DNA segment) as barcodes [S9]. In the simulation, we purposely choose the mass of a tag (m_{tag}) three times heavier than a normal monomer to replicate the tags used in the experiments. We proportionally increase the solvent friction of the tags $\Gamma_{tag} = 3\Gamma_i$. We use the Brownian dynamics to solve the equation of motion of a monomer i having a mass m_i and solvent friction Γ_i as

$$m_i \ddot{\vec{r}}_i = \vec{\nabla}_i \left[U_{LJ} + U_{FENE} + U_{bend} + U_{wall}\right] - \Gamma_i \vec{v_i} + \eta_i \tag{S5}$$

where $\Gamma_i = 0.7\sqrt{m_i \epsilon^2/\sigma^2}$ is the frictional coefficient arising from solvent-monomer interaction. For the case of a tag, $m_{tag} = 3m_i$ and $\Gamma_{tag} = 2.1\sqrt{m_i \epsilon^2/\sigma^2}$. The Gaussian white noise η_i arising from thermal fluctuation is delta correlated and expressed as $\langle \eta_i(t).\eta_j j(t') \rangle = 2dk_BT\Gamma\delta_{ij}\delta(t-t')$ with $d = 3$ in three dimensions. We express length and energy in units of σ and ϵ respectively such that $k_BT/\epsilon = 1.0$. The parameters for FENE potential in Eq. (S2) are κ_F and R_0, and set to be $\kappa_F = 30\epsilon/\sigma^2$ and $R_0 = 1.5\sigma$. The numerical integration of Eq. (S5) is implemented using the algorithm introduced by Gunsteren and Berendsen [S10]. Our previous experiences with BD simulation suggest that for a time step $\Delta t = 0.01$ these parameter values produce stable trajectories over a very long period of time and do not lead to an unphysical crossing of a bond by a monomer [S4, S5]. The average bond length stabilizes to $\langle b_l \rangle = 0.971 \pm 0.001\sigma$ with negligible fluctuation regardless of the chain size and rigidity [S4]. Hence we relate the polymer's contour length L and the number of monomers N as $L = (N-1)\langle b_l \rangle$.

II. SECTION 2: TENSION PROPAGATION AND THE SIMULATION MOVIE

Tension propagation plays an important role in governing a semi-flexible chain's motion through the double nanopore setup. The presence of heavier and bulkier tags attached in random positions along the chain's contour length makes the propagation of the tension non-uniform and leads to undulations in the velocity profile. Fig. S2 illustrates an example of such non-uniformity in the monomer velocity during a single scan. After the passage of a heavier tag T_8 through the right pore, the monomers behind T_8 travel at a higher velocity than T_8, and the monomers equidistance from the tags T_8 and T_7 roughly follow the average velocity of the chain (Fig. S2(a)). This faster motion of monomers continues uninterruptedly until the tension front reaches the next tag T_7 and then the motion of the segment tapers down to the velocity of T_7. This process continues for segments between any two tags throughout all the scanning events in both directions of translocations (Please watch the simulation movie for visualization).

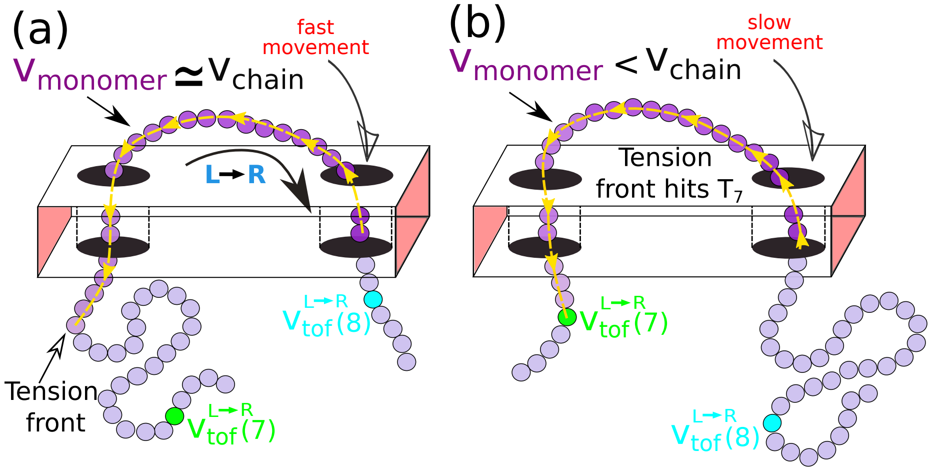

FIG. S2. Demonstration of tension propagation (TP) through the chain during a $L \rightarrow R$ translocation event. (a) The sudden onset of faster movement of monomers begins right after the passage of T_8 through the right nanopore and continues until the TP front hits the next tag T_7. The yellow dashed line represents the extent of the tensile force. The monomers affected by the tension front are depicted in a darker pink shade with fading colors that show the diminishing effect of tension propagation away from the pore. The velocity of the monomers is comparable to the average chain velocity v_{chain} in this case. (b) After a while, TP front reaches to T_7 and slower translocation begins due to the tag's inertia and larger viscous drag.

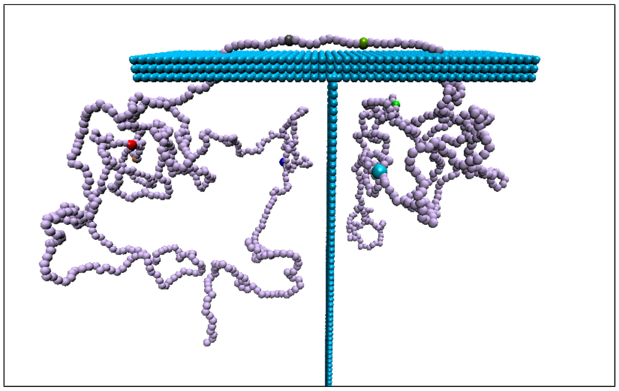

FIG. S3. A single movie frame (screen-shot) shows the translocation process of a dsDNA through the double nanopore setup. Monomers are depicted using pink beads and tags are colored with the color code following Fig. 1(b). The walls are constructed with repulsive LJ particles colored in .

[S1] Grest, G. S. & Kremer, K. Molecular Dynamics Simulation for Polymers in the Presence of a Heat Bath. Phys. Rev. A **33**, 3628–3631 (1986).
[S2] Binder, K. Monte Carlo and Molecular Dynamics Simulations in Polymer Science, Oxford University Press, Chap. 2 (1995).
[S3] Adhikari, R. & Bhattacharya, A. Driven Translocation of a Semi-Flexible Chain through a Nanopore: A Brownian Dynamics Simulation Study in Two Dimensions. The Journal of Chemical Physics **138**, 204909 (2013).
[S4] Huang, A., Bhattacharya, A. & Binder, K. Conformations, Transverse Fluctuations, and Crossover Dynamics of a Semi-Flexible Chain in Two Dimensions. The Journal of Chemical Physics **140**, 214902 (2014).
[S5] Huang, A., Adhikari, R., Bhattacharya, A. & Binder, K. Universal Monomer Dynamics of a Two-Dimensional Semi-Flexible Chain. EPL **105**, 18002 (2014).
[S6] Huang, A., Reisner, W. & Bhattacharya, A. Dynamics of DNA Squeezed Inside a Nanochannel via a Sliding Gasket. Polymers **8**, 352 (2016).
[S7] Bernier, S., Huang, A., Reisner, W. & Bhattacharya, A. Evolution of Nested Folding States in Compression of a Strongly Confined Semiflexible Chain. Macromolecules **51**, 4012–4022 (2018).
[S8] Landau, L. D., Lifshitz, E. M.Statistical Physics, Pergamon Press (1981).

[S9] Zhang, Y., Liu, X., Zhao, Y., Yu, J.-K., Reisner, W. & Dunbar, W. B. Single Molecule DNA Resensing Using a Two-Pore Device. Small **14**, 1801890 (2018).

[S10] van Gunsteren, W. F. & Berendsen, H. J. C. Algorithms for Brownian Dynamics. Molecular Physics **45**, 637–647 (1982).
